# Assessment of H-index and research impact amongst academic medical oncologists in Canada

**DOI:** 10.3389/frma.2026.1743565

**Published:** 2026-02-18

**Authors:** Sera Whitelaw, Daniel Jousé Guerra Ordaz, Amina Moustaqim-Barrette, Elena Netchiporouk, Ivan V. Litvinov

**Affiliations:** 1 Department of Medicine, University of Toronto, Toronto, ON, Canada; 2 Faculty of Medicine and Health Sciences, McGill University, Montreal, QC, Canada; 3 Division of Dermatology, McGill University, Montreal, QC, Canada; 4 St. Mary's Hospital, St. Mary's Research Centre, Montreal, QC, Canada

**Keywords:** academia, Canada, h-index, oncology, rank, research funding

## Abstract

**Introduction:**

The h-index is widely used to measure academic productivity in medicine, yet data on research output among academic medical oncologists in Canada remain limited. This study aimed to characterize the academic profiles of Canadian medical oncologists and identify factors associated with higher h-index scores.

**Methods:**

A cross-sectional analysis of medical oncologists affiliated with Canadian universities was conducted using publicly available faculty listings, Scopus Author IDs, and professional certification records. Key demographic and academic variables—including sex, years since certification, academic rank, graduate degrees, region of practice, and Canadian Institutes of Health Research (CIHR) funding—were collected. Univariate and multivariate log-linear regression models were used to assess the relationship between these factors and the h-index.

**Results:**

A total of 391 medical oncologists were identified across 15 Canadian institutions. The median national h-index was 14.0, with regional and sex-based differences noted in descriptive statistics. However, in adjusted analyses, only higher academic rank and receipt of CIHR funding were significantly associated with increased h-index values. Other variables, including sex, graduate degrees, region, and years of practice, were not significant predictors of academic productivity.

**Conclusion:**

These findings highlight the role of institutional advancement and research funding in supporting academic output and may guide future research, policy development, and evaluation practices in academic oncology.

## 1 Introduction

Academic productivity is a key measure of success in medical research, with the h-index serving as one of the most widely used metrics to assess the impact of a researcher's work. The h-index takes into account both the number of publications and the frequency with which they are cited (Hirsch, 2005, 2007). In academic medicine, the h-index is frequently used in faculty hiring, promotions and funding decisions, helping to quantify research contributions within a given specialty (Zaorsky et al., 2020; Saraykar et al., 2017; Wang et al., 2022).

Medical oncology is a rapidly evolving subspecialty marked by groundbreaking scientific advancements that is reshaping patient care. Medical oncologists play a crucial role in advancing cancer research, contributing to innovations such as developments in targeted therapies, immunotherapies, and personalized medicine that ultimately enhance patient outcomes (Krzyszczyk et al., 2018). However, despite the high volume of research output in medical oncology, there is limited data on the typical h-index values among academic medical oncologists and the factors influencing research productivity in this field.

Our recent study examined the h-index and factors associated with academic productivity among hematologists in Canada, highlighting key demographic and professional variables that may impact research output (Ordaz et al., 2024). Using a similar methodology, this study aims to investigate the h-index among medical oncologists across Canadian institutions. Our goals are to define the distribution of h-index values in medical oncology and identify variables associated with higher research productivity. By clarifying the academic landscape in medical oncology, this study may offer insights into research trends, inform institutional policies, and support initiatives to foster scholarly excellence within the field.

## 2 Materials and methods

### 2.1 Information sources

For this cross-sectional study, we identified 17 Canadian universities with Medical Oncology departments listed on their institutional websites. Between April 2024 and July 2024, we consulted faculty directories for each university to obtain the complete list of members. The full list of faculty members was publicly accessible for 15 universities and was thus included in this study. Since this study only used publicly available data, it was exempt from ethics review.

Faculty members included those with ranks of assistant professor, associate professor, and full professor. Only physicians holding a subspecialty diploma in medical oncology were part of the study. Members who did not hold an academic professorship, were not physicians (MD or equivalent), or were retired as of January 2024 were excluded. Data extraction was carried out by a single investigator (SW) and validated by a senior investigator (IVL).

The following data was collected for each faculty member: full name, sex, affiliated university region (Atlantic, Quebec, Ontario, Prairies, British Columbia), rank (assistant professor, associate professor, professor), years since obtaining the College des Medecins du Quebec (CMQ) and/or the Fellow of the Royal College of Physicians of Canada (FRCPC) certification, postgraduate degrees (MSc, PhD, other), recent Canadian Institutes of Health Research (CIHR) funding, and their h-index (based on Scopus Author ID search, completed July 2024).

A standardized approach was used to identify faculty members for all positions. This approach was utilized by the methodology used in our previous papers on academic dermatology and academic hematology (Ordaz et al., 2024; Azar et al., 2022). Our initial search used author's first and last names as they appeared on the faculty website in combination with their institutional affiliation. If this was unsuccessful, we then searched by last name and institutional affiliation. The results were verified by evaluating that published papers were relevant to medical oncology in their Scopus profile. We also searched for postgraduate degrees using department websites, LinkedIn, Google Scholar, as well as research articles and conference proceedings.

We searched the CMQ directory to determine the number of years since certification for Quebec medical oncologists. For non-Quebec medical oncologists, we used the FRCPC directory to determine the years since their certification. For Canadian medical oncologists without FRCPC certification, we analyzed LinkedIn reports to find the years since certification. If that information was not available, we searched institutional websites and the websites of provincial Colleges of Physicians and Surgeons. If medical oncologists had been practicing for ≥30 years (based on FRCPC certification or an equivalent), we used provincial physician directories to confirm they were still practicing.

We categorized the geographic location of the included universities into five different geographic region: Atlantic (Dalhousie University, Memorial University), Quebec (McGill University, Universite de Montreal), Ontario (McMaster University, Queens University, Western University, University of Ottawa, University of Toronto, Northern Ontario School of Medicine), Prairie Provinces (University of Alberta, University of Calgary, University of Manitoba, University of Saskatchewan), and British Columbia (University of British Columbia).

We used the CIHR database to identify members who have obtained research funding within the last 5 years. Author profiles on Scopus Author ID were used to obtain the h-index. If numerous author profiles were available, we used the highest related h-index.

### 2.2 Analysis

Descriptive statistics were first used to describe the dataset, stratified by geographical location. We presented continuous variables using median and ranges and categorical variables as counts and percentages. The main outcome variable of interest was h-index, with years since FRCPC certification or equivalent, academic rank, affiliated university geographical distribution, CIHR funding, graduate degree completion, and sex included in modeling as explanatory variables. All variables were cross-tabulated with the outcome variable and those with a *p*-value < 0.05, and/or which were deemed to be conceptually relevant, were included for further multivariate analysis. Variables were also assessed for multicollinearity. As previously described (Ordaz et al., 2024; Azar et al., 2022), as h-index can only take positive values, we elected to shift the h-index by one to the right and apply natural log transformation. With this transformation, taking the exponential of an estimated beta coefficient and subtracting 1 provided the percentage change in the adjusted H-index for a one-unit increase in the variable of interest, while accounting for the other covariates.

Postgraduate degree status was missing for approximately 58% of the cohort (Supplementary Table 1). Because missingness was plausibly related to observed characteristics such as academic rank, years since certification, and geographic region, we assumed data were missing at random and performed multiple imputation using chained equations (20 imputations). Degree was imputed and estimates were pooled using Rubin's rules. Multiple imputation preserves sample size and reduces bias under a missing-at-random assumption, whereas complete-case analysis may yield biased estimates and reduced precision when missingness is related to observed characteristics. Complete-case results are therefore presented as sensitivity analyses.

All analyses were conducted using R statistical programming version 4.4.3.

## 3 Results

A total of 391 medical oncologists across Canada were included in our study, with 169 individuals (43.2%) identified as females (Table 1). The median years since obtaining FRCPC or its equivalent was 16.0 years [range 2.0–46.0]. Regional differences were observed, with the shortest median interval in British Columbia (14.5 [range 3.0–40.0]) and the longest in Quebec (22.0 [range 3.0–36.0]). Ontario had the highest proportion of oncologists holding an M.Sc. (23.1%), and Ph.D. (17.2%) degrees. Faculty ranks varied geographically: British Columbia had the highest proportions of professors (19.1%) and associate professors (40.4%), while Quebec had the largest share of assistant professors (65.9%). Ontario also had the greatest proportion of CIHR-funded oncologists, representing 44.6% of awardees. The median h-index for the national cohort was 14.0 [range 0.0–112.0], with British Columbia showing the highest regional median at 17.0 [range 0.0–96.0]. These groups differed significantly, with a *p*-value < 0.001.

**Table 1:** Descriptive overview of oncology faculty members across Canadian by universities.

**Variable**	**ATL (*N* = 36)**	**BC (*N* = 47)**	**ON (*N* = 186)**	**PRAIRIE (*N* = 78)**	**QC (*N* = 44)**	**Total (*N* = 391)**	***p*-value**
Sex							0.009
Female	16 (44.4%)	31 (66.0%)	79 (42.5%)	26 (33.3%)	17 (38.6%)	169 (43.2%)	
Male	20 (55.6%)	16 (34.0%)	107 (57.5%)	52 (66.7%)	27 (61.4%)	222 (56.8%)	
H-Index							< 0.001
Count	33	46	178	77	44	378	
Median	5 [1.00–54.00]	17 [0.00–96.00]	16.5 [1.00–95.00]	14 [3.00–60.00]	17 [1.00–112.00]	14 [0.00–112.00]	
M.Sc.							0.015
Yes	2 (5.6%)	6 (12.8%)	43 (23.1%)	16 (20.5%)	7 (15.9%)	74 (18.9%)	
Ph.D.							0.038
Yes	2 (5.6%)	5 (10.6%)	32 (17.2%)	7 (9.0%)	6 (13.6%		
Other							0.008
Yes	1 (2.8%)	8 (17.0%)	22 (11.8%)	6 (7.7%)	0 (0.0%)	37 (9.5%)	
Rank							0.469
Assistant professor	19 (52.8%)	19 (40.4%)	100 (53.8%)	38 (48.7%)	29 (65.9%)	205 (52.4%)	
Associate professor	12 (33.3%)	19 (40.4%)	55 (29.6%)	27 (34.6%)	8 (18.2%)	121 (30.9%)	
Professor	5 (13.9%)	9 (19.1%)	31 (16.7%)	13 (16.7%)	7 (15.9%)	65 (16.6%)	
Years in practice							0.239
Count	36	44	181	74	39	374	
Median	17 [5.00–42.00]	14.5 [3.00–40.00]	16 [2.00–46.00]	17 [3.00–43.00]	22 [3.00–36.00]	16 [2.00–46.00]	
CIHR funding							0.111
Yes	8 (22.2%)	19 (40.4%)	83 (44.6%)	27 (34.6%)	16 (36.4%)	153 (39.1%)	

CIHR, Canadian Institutes of Health Research; BC, British Columbia; ATL, Atlantic Canada; ON, Ontario; PRAIRIE, Prairie provinces.

**Figure 1:**
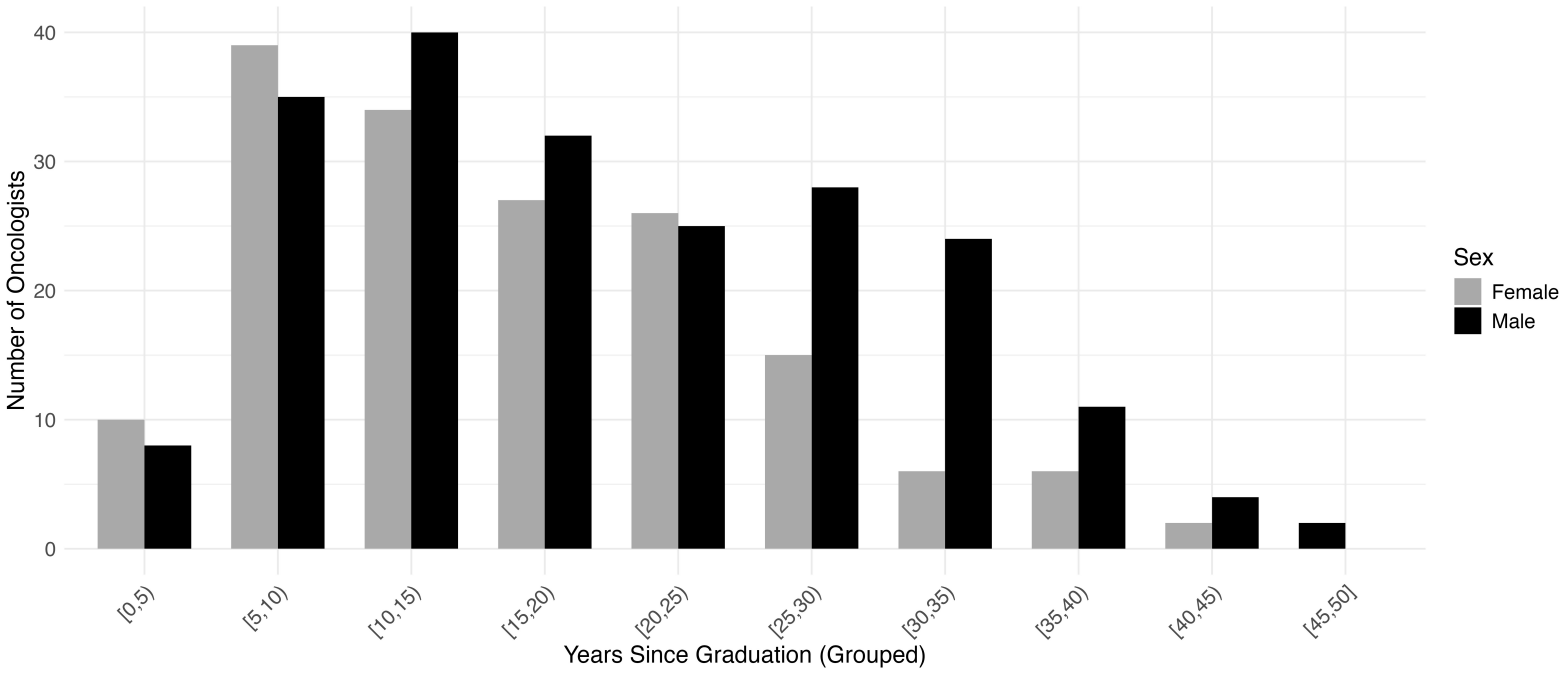
Distribution of years since Fellow of the Royal College of Physicians of Canada (FRCPC) certification or equivalency among females (*n* = 165) and males (*n* = 209).

**Figure 2:**
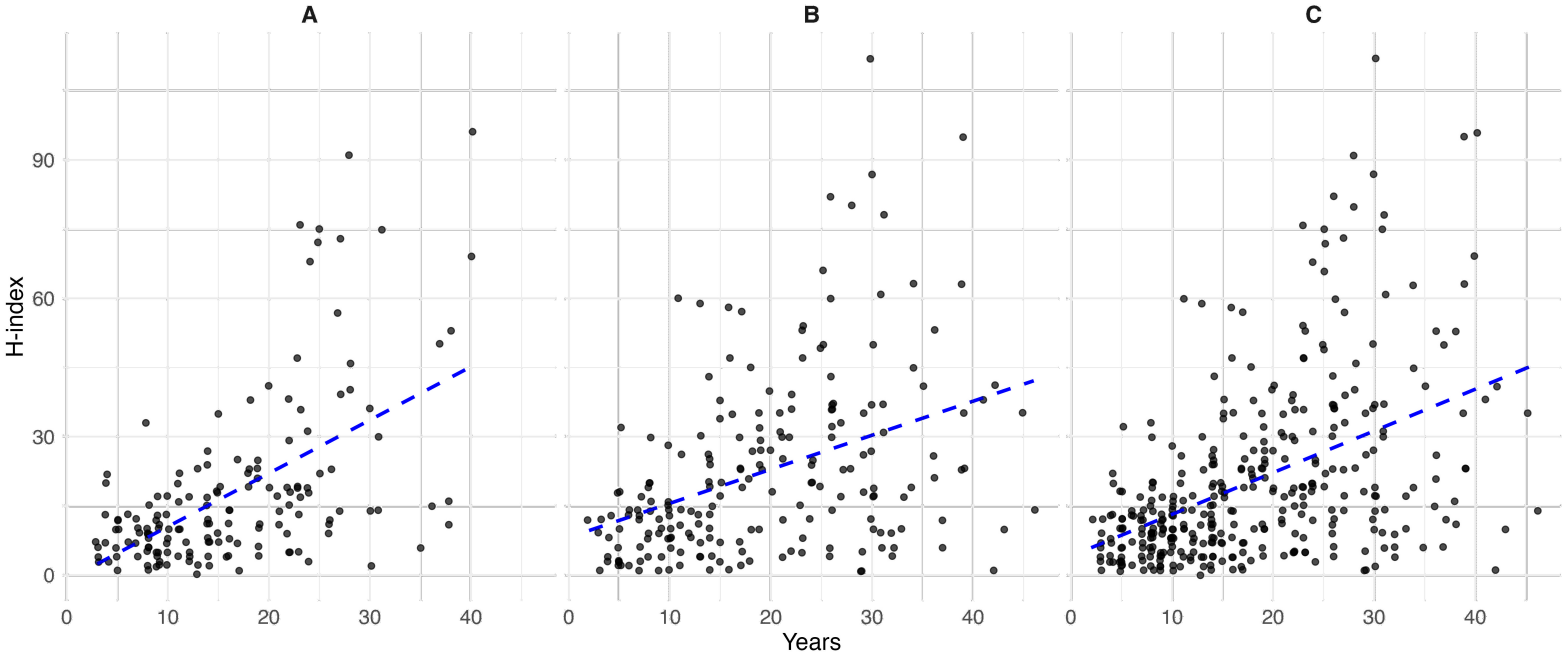
Years since Fellow of the Royal College of Physicians of Canada (FRCPC) certification or equivalency by h-index in **(A)** females (*n* = 165), **(B)** males (*n* = 209) and **(C)** overall (*n* = 374).

**Figure 3:**
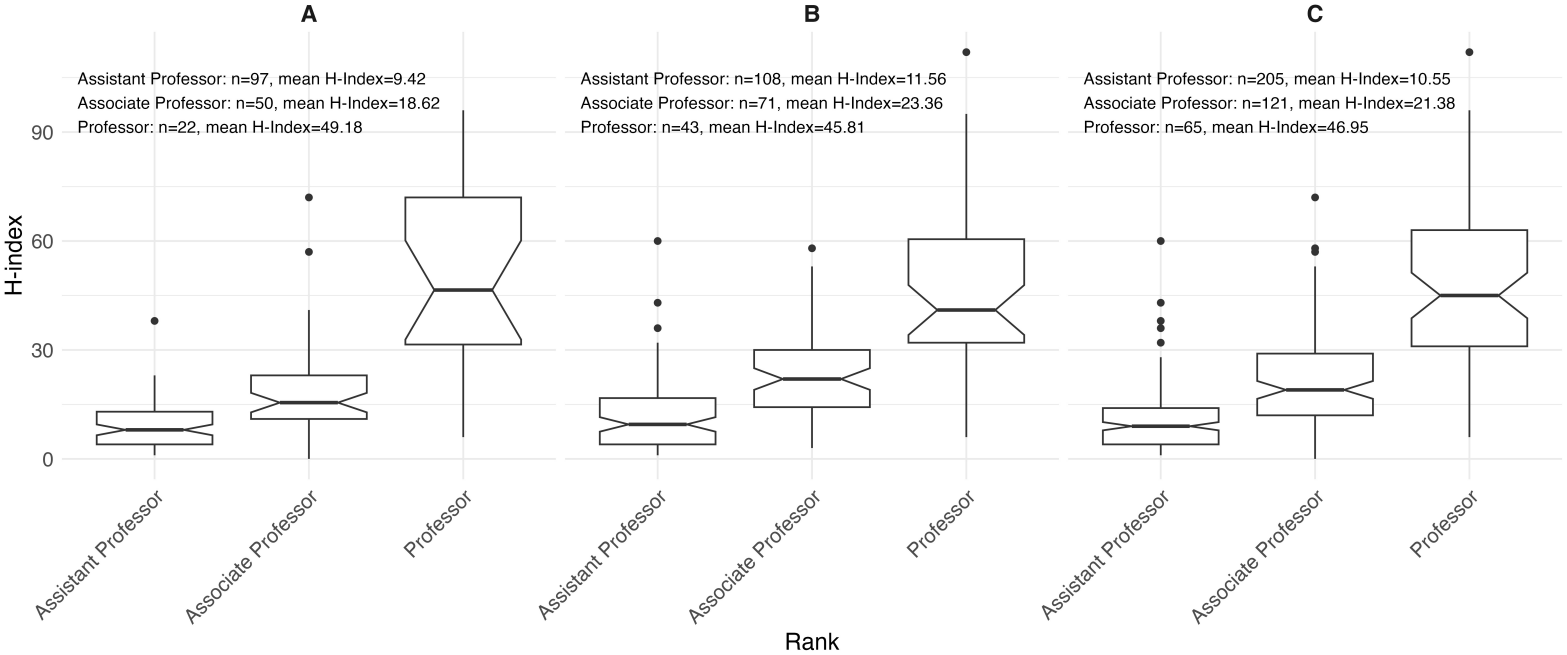
Distribution of h-index by academic rank among **(A)** females (*n* = 169), **(B)** males (*n* = 222), and **(C)** overall (*n* = 391) for both sexes.

**Figure 4:**
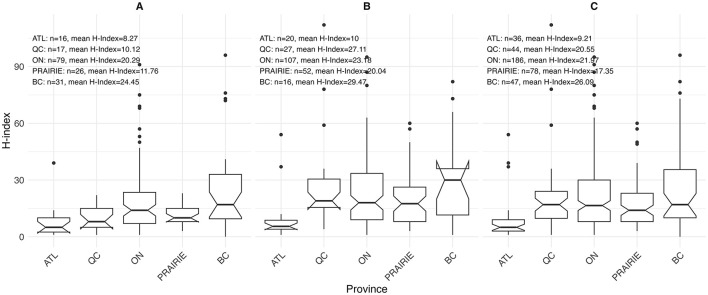
Distribution of h-index by territory among **(A)** females (*n* = 169), **(B)** males (*n* = 222), and **(C)** overall (*n* = 391) for both sexes.

**Figure 5:**
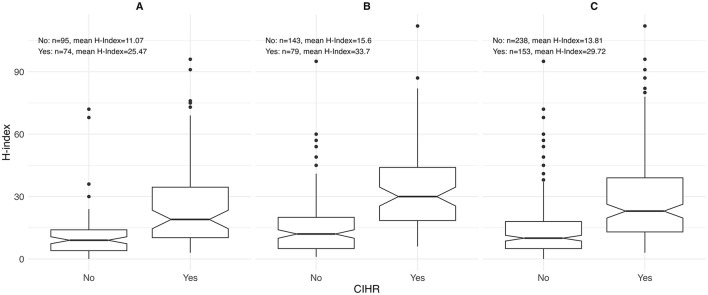
Distribution of h-index by receipt of Canadian Institutes of Health Research (CIHR) funding among **(A)** females (*n* = 169), **(B)** males (*n* = 222), and **(C)** overall (*n* = 391) for both sexes.

**Figure 6:**
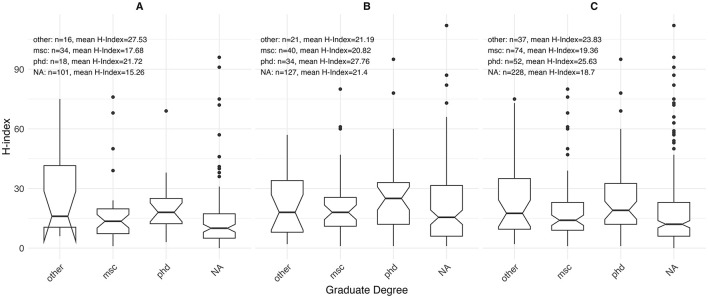
Distribution of h-index by graduate degree status among **(A)** females (*n* = 169), **(B)** males (*n* = 222), and **(C)** overall (*n* = 391).

**Figure 7:**
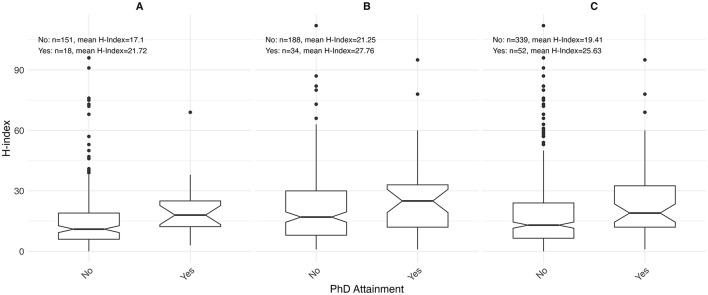
Distribution of h-index by Ph.D. status among **(A)** females (*n* = 169), **(B)** males (*n* = 222), and **(C)** overall (*n* = 391) for both sexes.

Sex-stratified descriptive statistics are presented in Table 2. Female oncologists had a significantly shorter time since certification (14.0 years [range 3.0–40.0]) compared to their male counterparts (18.0 years [range 2.0–46.0]), with a *p*-value of 0.005. This pattern is also illustrated in Figure 1, where the highest concentration of female oncologists falls between 5 and 10 years since certification, whereas male oncologists most commonly fall between 10 and 15 years. There is a significant difference in the h-index between sexes, with males having a higher h-index (17.0 [range 1.0–112.0]) than females (12 [range 0.0–96.0]), *p* = 0.003. Figure 2 shows that a higher number of males have elevated h-index scores early in their careers compared with females. It also illustrates that females exhibit a steeper increase in the h-index over time. Similarly, a significant difference exists in the distribution of sex across territories, *p* = 0.009, which can be noted in Figure 3. However, no significant sex-based differences were observed in graduate degrees held, professorial rank, or CIHR funding status. Figures 4–7 illustrate that males and females show similar patterns across these variables.

**Table 2:** Descriptive overview of oncology faculty members across Canadian by sex.

**Variable**	**Female (*N* = 169)**	**Male (*N* = 222)**	**Total (*N* = 391)**	***p*-value**
H-Index				0.003
Count	163	215	378	
Median	12 [0.00–96.00]	17 [1.00–112.00]	14 [0.00–112.00]	
Territory				0.009
ATL	16 (9.5%)	20 (9.0%)	36 (9.2%)	
BC	31 (18.3%)	16 (7.2%)	47 (12.0%)	
ON	79 (46.7%)	107 (48.2%)	186 (47.6%)	
PRAIRIE	26 (15.4%)	52 (23.4%)	78 (19.9%)	
QC	17 (10.1%)	27 (12.2%)	44 (11.3%)	
M.Sc.				0.804
No	101 (74.8%)	127 (76.0%)	228 (75.5%)	
Yes	34 (25.2%)	40 (24.0%)	74 (24.5%)	
Ph.D.				0.202
No	101 (84.9%)	127 (78.9%)	228 (81.4%)	
Yes	18 (15.1%)	34 (21.1%)	52 (18.6%)	
Other degree				0.905
No	101 (86.3%)	127 (85.8%)	228 (86.0%)	
Yes	16 (13.7%)	21 (14.2%)	37 (14.0%)	
Rank				0.142
Assistant professor	97 (57.4%)	108 (48.6%)	205 (52.4%)	
Associate professor	50 (29.6%)	71 (32.0%)	121 (30.9%)	
Professor	22 (13.0%)	43 (19.4%)	65 (16.6%)	
Years in practice				0.005
Count	165	209	374	
Median	14 [3.000–40.00]	18 [2.00–46.00]	16 [2.00–46.00]	
CIHR Funding				0.1
No	95 (56.2%)	143 (64.4%)	238 (60.9%)	
Yes	74 (43.8%)	79 (35.6%)	153 (39.1%)	

Log-linear regression models were employed to further clarify the effects of covariates on the h-index (Table 3). Academic rank, obtaining CIHR funding, and region of practice influenced the academic productivity of oncologists. Higher academic ranks were associated with higher h-indices, with associate professors (1.34 [95% CI 1.16–1.54], *p* < 0.001) and full professors (1.69 [95% CI 1.44–1.97], *p* < 0.001) outperforming assistant professors. Obtaining CIHR funding was a predictor of a higher h-index (1.35 [95% CI 1.19–1.53], *p* < 0.001). Among regions, only ATL was a statistically significant predictor of h-index (0.71 [95% CI 0.52–0.95], *p* = 0.024). After adjusting for all factors, the multivariate analysis showed that none of the covariates had a significant influence on academic productivity. A Multivariate Imputation by Chained Equations (MICE) further demonstrated that academic rank and receipt of CIHR funding significantly impacted academic productivity. Consistent with the univariate analysis, seniority positively affected academic productivity, with associate professors (1.22 [95% CI 1.05–1.42], *p* = 0.010) and full professors (1.41 [95% CI 1.15–1.73], *p* < 0.001). Receipt of CIHR funding also influenced productivity (1.23 [95% CI 1.08–1.40], p = 0.002). Sex, postgraduate degree, years in practice, and region of practice did not affect the productivity of medical oncologists.

**Table 3:** Results of the log-linear univariate and multivariate analyses.

	**Unadjusted**				**Adjusted**				**Imputed dataset (MICE)**			
**Variable**	**ExpBeta**	**Lower bound**	**Upper bound**	***p*** **-value**	**ExpBeta**	**Lower bound**	**Upper bound**	***p*** **-value**	**ExpBeta**	**Lower bound**	**Upper bound**	***p*** **-value**
**Sex**												
Female	Reference				Reference				Reference			
Male	1.09	0.97	1.24	0.160	1.03	0.85	1.27	0.744	1.05	0.924	1.19	0.456
**Degree**												
M.Sc.	Reference				Reference				Reference			
Ph.D.	1.12	0.90	1.38	0.305	1.07	0.86	1.33	0.551	1.06	0.92	1.22	0.439
Other	1.06	0.83	1.34	0.645	1.03	0.80	1.31	0.830	1.04	0.89	1.22	0.587
**Rank**												
Assistant professor	Reference				Reference				Reference			
Associate professor	1.34	1.16	1.54	< 0.001	1.19	0.92	1.54	0.181	1.22	1.05	1.42	0.010
Professor	1.69	1.44	1.97	< 0.001	1.39	0.98	1.97	0.063	1.41	1.15	1.73	< 0.001
**Years in practice**												
Count	1.01	1.01	1.02	< 0.001	1.00	0.99	1.02	0.547	1.01	1.00	1.01	0.120
**Region**												
QC	Reference				Reference				Reference			
ATL	0.71	0.52	0.95	0.024	0.613	0.28	1.22	0.191	0.75	0.56	1.00	0.054
QC	1.03	0.80	1.32	0.801	1.031	0.65	1.65	0.898	1.00	0.77	1.29	0.986
ON	1.00	0.82	1.22	0.996	0.987	0.69	1.45	0.945	0.97	0.79	1.19	0.740
Prairies	0.98	0.78	1.23	0.854	0.989	0.66	1.52	0.957	0.97	0.77	1.22	0.786
**CIHR Funding**												
No	Reference				Reference				Reference			
Yes	1.35	1.19	1.53	< 0.001	1.16	0.951	1.42	0.143	1.23	1.079	1.40	0.002

## 4 Discussion

In this study, we describe the characteristics of Canadian medical oncologists and evaluate their influence on the h-index. We identify two factors associated with a higher h-index: academic seniority and CIHR funding. Sex, years since FRCP certification, graduate degrees, and region did not have a significant impact on academic productivity after multivariate analysis.

Our study aligns with previous research conducted by our group in academic hematology and dermatology, where the h-index increases with academic seniority, as expected (Ordaz et al., 2024; Azar et al., 2022). Here we report curves that are specific to this specialty. Other studies involving various medical and surgical specialties have similar findings (Shanmugasundaram et al., 2023; Rad et al., 2010; Lopez et al., 2014; Ashfaq et al., 2018; Semaan et al., 2016). The specific details to each specialty may be useful in promotion committees when evaluating candidates for academic advancement. Using the h-index as a metric to support the promotion of junior faculty members can be a reliable tool to enhance the assessment of new candidates (Wang et al., 2022).

Similar to our previous studies on academic hematology and dermatology, CIHR funding positively influenced the h-index (Ordaz et al., 2024; Azar et al., 2022). Other studies across various specialties have shown that securing funding from the National Institutes of Health or the National Institute of Mental Health positively influences the h-index (Saraykar et al., 2017; Rezek et al., 2011; Svider et al., 2014; Colaco et al., 2013; Svider et al., 2012). The link between governmental funding and a higher h-index may indicate that more academically productive individuals are more effective at securing research grants.

The influence of sex on the h-index remains unclear in the literature. In our study, we observed sex-based differences in descriptive analyses. However, these differences were no longer significant after univariate and multivariate adjustments, suggesting that sex is not an independent determinant of the h-index. Similarly, several other multivariate analyses have shown that sex is not a predictor of the h-index (Azar et al., 2022; Shanmugasundaram et al., 2023). On the other hand, some studies suggest that sex influences the h-index (Paik et al., 2014; Mayer et al., 2017), even when accounting for various factors such as academic rank (Ordaz et al., 2024; Lopez et al., 2014). A systematic review and meta-analysis led by Ha et al. (2021) demonstrated the influence of sex on the h-index. The influence of sex on the h-index may vary across specialties, with women's h-index being lower in male-dominated fields. A lack of same-sex mentors, role models, and opportunities early in their careers might negatively affect women's academic progress (Zhuge et al., 2011; Jagsi et al., 2006; Reed et al., 2011).

Our study found that a graduate degree did not affect the h-index. Similarly, other studies have shown that graduate degrees do not influence the h-index (Svider et al., 2014; Monir et al., 2020). However, other studies suggest a positive impact of a graduate degree on the h-index, or the 5-year-h-index (Ordaz et al., 2024; Azar et al., 2022; Keough et al., 2020).

In the descriptive analysis, a notable difference in the h-index was observed across regions. However, in the univariate analysis, only the ATL region showed a significantly lower h-index compared to Quebec (0.71 [95% CI 0.52–0.95], *p* = 0.024). After adjusting for other covariates in the multivariate model, this difference was no longer statistically significant, but it remained close to the threshold of significance (0.75 [95% CI 0.56–1.00]), *p* = 0.054. Our recent study on hematology found that BC and ON regions had higher h-indices than QC, but no significant difference was observed with ATL (Ordaz et al., 2024). The regional variation in h-index is an important observation. Further research should be conducted to identify the root causes of these differences. Potential reasons may include disparities in research infrastructure, institutional support, academic culture within the center, and access to funding.

The use of the h-index has its own limitations. This metric does not consider the authorship position, thus overlooking the academic contribution of each co-author. First authors, senior authors, and middle authors are treated equally, despite the different levels of intellectual input usually associated with these roles (Wang et al., 2022). The h-index often benefits senior researchers, as it tends to grow over time through the accumulation of publications and citations. Older works may continue to accumulate citations even if there is no ongoing research activity, enabling senior academics to maintain or increase their h-index regardless of current productivity (Mondal et al., 2023; Bornmann and Daniel, 2007; Rousseau and Leuven, 2008). Additionally, the h-index does not account for the quality of the publication and can be affected by excessive self-citation, which can artificially manipulate the metric (Mondal et al., 2023; Bartneck and Kokkelmans, 2010; van Raan, 2006). The language of publication may influence the h-index, as articles published in English tend to garner more citations and are more visible in search engine results (Di Bitetti and Ferreras, 2016; Rovira et al., 2021). Finally, the h-index may underestimate or overestimate the academic impact of researchers with well-known names, as their publications might not be properly attributed (Costas and Bordons, 2007).

In response to the limitations of using indicators like the h-index, the San Francisco Declaration on Research Assessment (DORA) was created. It advocates for evaluating an individual's scientific contribution rather than relying solely on publication metrics when making decisions about funding, hiring, tenure, or promotion. While metrics can serve as evidence of impact, they should not replace qualitative assessment of research contributions (San Francisco Declaration on Research Assessment (DORA), 2025).

It is important to acknowledge the limitations of this study. Despite our efforts to gather information from multiple platforms, the h-index and years since practice were not always available. However, sensitivity analyses for the independent variables were conducted and produced similar results to those from the complete dataset. One limitation is the reliance on sex-based data. Gender information was not readily accessible; therefore, this study is based on sex without considering the diversity of the gender spectrum. Additionally, the oncology faculty members were identified from online faculty lists, which may not reflect real-time information. Despite our attempts to exclude recently retired and inactive oncologists, some may have been retained. Likewise, recently recruited members might not be included, as the list may not have been updated. Finally, individual information such as ethnic background, protected research time, non-governmental funding, and institutional support levels was not readily available. To gain a better understanding of academic medical oncology, further research should include specific questionnaires to assess factors like leave of absence, protected research time, and non-governmental grants, which are not easily found online. Nevertheless, we believe this study accurately characterizes the features of academic medical oncologists in Canada and the impact of each characteristic on the h-index.

In conclusion, this study describes the characteristics of academic medical oncologists in Canada. It highlights that a higher h-index is linked to a higher academic rank and the receipt of CIHR funding. Sex, region of practice, graduate degree, and years of practice did not affect the h-index.

## Data Availability

Publicly available datasets were analyzed in this study. This data can be found here: The datasets were compiled from publicly available sources (faculty listings, Scopus Author IDs, and certification records). https://www.cmq.org/fr. https://www.scopus.com/. https://www.royalcollege.ca/en/directory/search.html. https://cihr-irsc.gc.ca.
