# Supplementary material for: Assessment of H-index and research impact amongst academic medical oncologists in Canada
Source: Front Res Metr Anal. 2026 Feb 18;11:1743565. doi: 10.3389/frma.2026.1743565 (PMC12958834; doi:10.3389/frma.2026.1743565)
Supplement: Supplementary file 1 [file Table_1.docx]

| **Supplementary Table 1 – Missingness of data** | | |
| --- | --- | --- |
| **Variable** | **Missing (N)** | **Missing (%)** |
| Degree | 228 | 58.3 |
| Years | 17 | 4.3 |
| H-index | 13 | 3.3 |
| Sex | 0 | 0.0 |
| Rank | 0 | 0.0 |
| Territory | 0 | 0.0 |
| CIHR funding | 0 | 0.0 |
